# Family Matters: The Role of Internalized Stigma in Shaping Burden Across Caregivers and Patients with Major Depression

**DOI:** 10.1192/j.eurpsy.2026.10211

**Published:** 2026-07-31

**Authors:** C. Toni, S. Cipolla, B. Della Rocca, A. Boiano, E. Corvino, P. Catapano, M. Di Vincenzo, V. Giallonardo, M. Luciano, A. Fiorillo

**Affiliations:** Psychiatry, University of Campania “L. Vanvitelli”, Naples, Italy

## Abstract

**Introduction:**

Major depressive disorder (MDD) carries a significant burden for both patients and families. Among psychosocial factors, internalized stigma worsens outcomes, self-perception, and social functioning. Yet, its impact on family burden and patient–caregiver dynamics is less explored.

**Objectives:**

This study primarily aimed to examine the role of depressive symptoms, quality of life (QoL), and internalized stigma in predicting patient and family burden. Secondarily, we aimed to assess the association between family coping strategies and the level of patient internalized stigma.

**Methods:**

We recruited 41 family units across 23 Italian centers, each including one patient with DSM-5 MDD and at least one family member. Participants completed questionnaires on socio-demographic and clinical data, psychopathology (stigma, QoL), caregiver burden, and coping. Regression analyses were run on significant predictors.

**Results:**

Regression analyses showed that depressive symptoms (beta= 0.16, p < 0.05), poorer QoL (beta= -0.38, p < 0.001), and higher internalized stigma (beta= 0.25, p < 0.001) significantly predicted greater objective burden in patients. When subjective burden was considered as the outcome, additional predictors included lower educational (beta= -0.17, p < 0.001) and longer illness duration (beta= 0.09, p < 0.05), alongside depressive symptoms (beta= 0.22, p < 0.01), QoL (beta= -0.24, p < 0.001), and internalized stigma (beta= 0.29, p < 0.001). Regarding family burden, both objective and subjective levels were strongly influenced by the patient’s internalized stigma (beta= 0.13, p < 0.05 and beta= 0.18, p < 0.001 respectively), underscoring its spillover effects on caregivers. Moreover, family coping strategies emerged as relevant correlates: higher levels of resignation among relatives (beta= 0.24, p < 0.001) predicted greater internalized stigma scores in patients.

**Conclusions:**

Our findings demonstrate that internalized stigma plays a pivotal role in shaping the burden of MDD, not only by amplifying patient difficulties but also by extending its negative impact to family members. Importantly, stigma operates partly beyond the illness itself, representing an independent psychosocial determinant of both patient and family distress. At the same time, stigma is not immutable: maladaptive coping patterns, such as resignation among relatives, may exacerbate it, whereas targeted interventions could help mitigate it. These results highlight the clinical relevance of psychoeducational and family-based programs, which may indirectly reduce patient internalized stigma, alleviate caregiver burden, and ultimately improve psychosocial outcomes in MDD.

**Disclosure of Interest:**

None Declared

